# Cardiac health knowledge and misconceptions among nursing students: implications for nursing curriculum design

**DOI:** 10.1186/s12912-017-0241-3

**Published:** 2017-08-15

**Authors:** Susan Ka Yee Chow, Yuen Yee Chan, Sin Kuen Ho, Ka Chun Ng

**Affiliations:** 1 School of Nursing, Tung Wah College, 31Wylie Road, Homantin, Kowloon, Hong Kong; 2 Queen Elizabeth Hospital, Kowloon, Hong Kong; 3 Registered Nurse, Master of Nursing, Kowloon, Hong Kong; 4 Tseung Kwan O Hospital, Kowloon, Hong Kong

**Keywords:** Cardiac knowledge, Cardiac misconceptions, Nursing students

## Abstract

**Background:**

Cardiac misconceptions are common among healthcare professionals. The development of professional knowledge is considered an essential component of nursing education. Nurses, regardless of their grade, skills, and experience, should be updated with information so as to be able to rectify their misconceptions, as these could affect patient health outcomes. As the literature evaluating the cardiac knowledge and misconceptions of nursing students is sparse, a study of the subject seems warranted.

**Methods:**

A cross-sectional sample survey was used to study the cardiac knowledge and cardiac misconceptions of nursing students in Hong Kong. The study sample included 385 senior nursing students from three universities. Their level of knowledge of cardiac disease was assessed using the modified Coronary Heart Disease Knowledge Test. The York Cardiac Beliefs Questionnaire (YCBQv1) was used to examine cardiac misconceptions.

**Results:**

The scores for the nursing students’ level of knowledge were diverse. Their mean score in the Cardiac Knowledge Test was 12.27 out of 18 (SD 2.38), with a range of 2–17. For cardiac misconceptions, their mean score in the YCBQv1 was 6.98 out of 20 (SD 2.84), with a range of 0–14. A negative correlation, *r* = −0.33 was found among students with more knowledge and fewer misconceptions. (*p* < 0.001). The Chi-square tests found some associations between the students’ experiences of caring for cardiac patients and misconceptions about stress and physiology.

**Conclusions:**

The results of our analyses indicate a diversity in levels of knowledge among the nursing students. Students with higher scores in cardiac knowledge did not necessarily have fewer misconceptions. There were associations between the students’ misbeliefs and their caregiving experiences with cardiac patients. This study presents a framework for designing the contents of cardiac nursing programmes and is a starting point for promoting research on misconceptions held by undergraduate nursing students. A new paradigm of teaching should include inputs from both perspectives to help students to make critical use of theoretical knowledge to rectify their misconceptions and pursue excellence in the working world.

## Background

Heart disease is an umbrella term used to describe Coronary Artery Disease, Myocardial Infarction, Angina, Atrial Fibrillation, Heart Rhythm Disorders, Cariomyopathy and Heart Failure, and other ailments [1]. According to the Centers for Disease Control and Prevention, Coronary Artery Disease is the most common type of heart disease and could lead to heart attacks [2]. Different nations have been developing strategies to reduce the risk of heart disease in their populations.

Nurses are expected to play an essential role in health promotion through delivering health education to patients and the general public. Cardiac knowledge is not solely about anatomy and physiology, but also encompasses patients’ self-management skills, medications, risk factors, exercise, stress, and diet [3]. Nurses, regardless of their grade, skills, and experience, should be updated with information so as to be able to rectify patients’ misconceptions about their condition and educate them about appropriate lifestyle changes [4].

Cardiac misconceptions refer to false or mistaken views, opinions, or attitudes about heart problems that can influence patients’ interpretations of the recovery journey and their coping strategies. The negative impacts include higher levels of emotional distress, slower recovery, and poor physical functioning, all of which affect the wellbeing of the patients [5]. A common cardiac misconception relates to the avoidance of stress. It is perceived to be a danger for patients with heart disease to get into arguments with people [6]. Other misconceptions include the view that people with heart problems must never get excited, and that having heart problems is a sign that the heart is worn out [7]. As healthcare workers spend a considerable amount of time with patients, they could introduce such misconceptions to patients or reinforce already held misconceptions, which would adversely affect patient health outcomes. There is a need for nurse clinicians to recognize that illness perceptions and cardiac misconceptions can have an impact on patients [8, 9].

Studies have been conducted on the knowledge and misconceptions of cardiac disease among different populations. A recent study reported significant variations in cardiac misconceptions between different groups of healthcare personnel, with medical and nursing students having higher misconception scores than graduated staff [8]. When comparing the maladaptive beliefs about heart disease held by nurses, nursing students, and patients with the disease, it was found that nursing students had fewer misconceptions than graduated nurses, while patients held significantly more misconceptions. As for patients with myocardial infarction, a common misconception is that they must avoid stress and excitement in their daily lives [6]. In a community sample of healthy adults, people who were older, male, or had not attained an academic degree were found to have more misconceptions [10]. A study conducted among the Amish found that they valued good health, which they believe is a gift from God. They do not believe that lifestyle changes can prevent cardiovascular disease, as preventive health is not a priority in the Amish culture [11]. When assessing the maladaptive beliefs about heart disease of patients in two countries, the Taiwanese had more misconceptions than their British counterparts. The Chinese population perceived stress to be one of the main causes of heart disease [12]. When people with chronic illnesses and people with coronary heart diseases were compared, the two groups of patients held similar cardiac misconceptions related to stress and excitement. This might be due to a misinterpretation of health education messages or to a lack of information from healthcare providers [13].

With regard to nurses’ knowledge of how to manage patients with cardiac diseases, it was found that home care nurses do not have sufficient grounding in evidence-based education to manage patients with heart failure [14]. A national survey conducted in Denmark showed that senior nurses, such as head nurses, were more familiar with evidence-based cardiac nursing than frontline nurses, as they read scientific journals more frequently. It should be noted that all nurses, regardless of their qualifications and rank, have the professional responsibility to keep abreast of recent healthcare research related to heart failure [15]. In China, a sample of hospital nurses, nursing faculty members, and nursing students were able to answer most of the questions that were posed on risk factors related to cardiovascular disease. Nevertheless, only a few of the respondents were able to correctly identify isolated systolic hypertension as a risk factor for cardiovascular disease. The nursing students were found to have inadequate knowledge to care for patients at risk of developing cardiovascular diseases [16].

While there are published studies on cardiac knowledge and cardiac misconceptions among healthcare and population groups [7, 8, 14–16], the literature relating to the knowledge and misconceptions of nursing students is sparse. The development of professional knowledge is considered an essential component of nursing education [17]. With regard to nursing education in colleges and universities, apart from providing factual and theoretical knowledge, nurse educators need to address the issue of cardiac misconceptions to ensure that future nurses are disseminating accurate information to meet the needs of patients. Although there is some correlation between cardiac knowledge and misconceptions, the constructs in the messages that are conveyed are different. Information on the relationship between knowledge and misconceptions among nursing students is limited, and little research has been conducted on the correlations and disparities between the two. Therefore, it is important to develop a better understanding of their association.

The research question of the study was “Is there any relationship between cardiac knowledge and misconceptions among nursing students”?

The objectives of this study are to: (1) examine the level of knowledge of nursing students with regard to cardiovascular diseases, (2) examine the misconceptions of nursing students with regard to cardiovascular diseases, (3) assess the relationship between the level of knowledge and the misconceptions of nursing students with regard to cardiovascular diseases, and (4) assess whether these misconceptions are associated with demographic characteristics.

## Methods

A cross-sectional sample survey was used to study the cardiac knowledge and cardiac misconceptions of nursing students in Hong Kong.

### Sample size and sampling

The sample size was estimated according to the equation designed by Rea [18].

$$ \frac{Z_a^2(.25)(N)}{Z_a^2(.25)+\left(N-1\right){ME}_p^2} $$

The calculation was based on the size of the population of senior-year nursing students in the three universities, which was about 660. The authors decided that given a 95% confidence level with a margin of error that did not exceed ±5%, a sample size of 243 would be required for a population of 660.

In Hong Kong, there are three publicly funded universities and three private institutions that offer a bachelor’s degree in nursing. The study sample included 385 students from the public universities. The criteria for inclusion in this study were: (1) senior year students who had completed the theoretical components on cardiovascular nursing and (2) had attended a clinical practicum in a medical unit. The rationale for the latter criterion was to ensure that the students had exposure to caring for patients with cardiovascular diseases.

### Research instruments

The authors developed a questionnaire on demographics to examine the personal particulars and the clinical learning experiences of students in cardiovascular nursing. Their level of knowledge on cardiac diseases was assessed using the modified Coronary Heart Disease Knowledge Test [19]. This measurement scale consists of 20 multiple choice questions that examine the respondent’s knowledge of coronary heart disease and the associated risk factors, which include physiology, body weight, stress, and exercise. The total score ranges from 0 to 20, with a higher score indicating a higher level of knowledge. Scores lower than 16 are considered to be low scores, and scores of 16 or higher are considered to be high scores. The scale was originally developed in 1991, and has acceptable validity and reliability [20]. The modified scale has been used widely among different population groups and various clinical environments and communities in the past two decades [21–24]. To assess cardiac misconceptions, the York Cardiac Beliefs Questionnaire (YCBQv1) was used. The tool consists of 24 items, with responses indicating agreement of disagreement. A higher score means a greater number of misconceptions. The scale demonstrates good content validity, good internal reliability, and test-retest stability. It was derived from two previous versions of misconception questionnaires and the items were drawn from statements made by patients living in Britain regarding their journey of living with a heart condition [25, 26]. The YCBQv1 has been translated into Chinese and used among different patient groups, including cardiac patients and people with chronic illnesses [7].

Due to cultural differences, the researchers conducted a test of the content validity and reliability of the Coronary Heart Disease Knowledge Test and the YCBQv1. The review panel consisted of four experts, including three experienced nurse educators and a physician. With regard to the knowledge test, two experts commented that two questions were irreverent, as they could not be used to identify knowledge of the risk factors for cardiovascular disease. The content validity of the scale was 0.92, with a total of 18 items that were rated as very relevant and relevant to the objectives of the study. For YCBQv1, four items were rated as somewhat relevant and not relevant by at least two members of the panel. The content validity score was 0.95, with a total of 20 remaining items. The above Content Validity Index was considered acceptable as an indicator of content validity [27]. With regard to the reliability test, 20 nursing students were invited to participate in examining the test-retest reliability. The same questionnaire was given to the participants two weeks apart. The Intra-class correlation (ICC) for the Coronary Heart Disease Knowledge Test was 0.81 and the ICC of the YCBQv1 was 0.93. The test-retest reliability of the two scales was considered appropriate, as ICC values of above 0.75 are indicative of good reliability [28].

### Data collection and responses

Data collection was conducted over a three-month period. Convenience sampling was used to collect data. The questionnaires were distributed to all senior-year nursing students with the assistance of the lecturers during lectures in one university, and through the social networks of the researchers in the other two universities. Three hundred and eighty-five questionnaires were sent out and 362 of them were returned, for a response rate of 94.02%. Twenty questionnaires were discarded because of the large amount of missing data and invalid responses. A total of 342 valid questionnaires were used for the data analysis.

### Data analysis

Statistical analyses were performed using IBM SPSS Statistics for Windows, Version 23.0 (IBM Corp, Armonk, NY). The demographic data and the scores on cardiac knowledge and misconceptions were examined using descriptive analysis. The correlation between cardiac knowledge and misconceptions was calculated using Pearson’s correlation. The association between the demographic data and individual items on misconception were examined using a Chi-square test. The level of significance for the two-tailed test was set at *p* < 0.05.

## Results

### Characteristics of the students

With regard to the demographics of the students, the majority of the students were single and did not have any cardiovascular diseases. A large majority (86.3%) of the students have been the primary caregiver of family members or relatives, while more than half of them have been the primary caregiver for clients with cardiovascular diseases during their clinical practicum (Refer to Table 1).

**Table 1:** Demographic characteristics (*N* = 342)

Variable	N	Percent (%)
Gender		
Male	96	28.1
Female	246	71.9
Marital Status		
Single	339	99.1
Married	3	0.9
Co-habiting	0	
Have had a cardiovascular disease		
Yes	332	97.1
No	10	2.9
Are the primary caregiver of family members or relatives with cardiovascular diseases		
Yes	295	86.3
No	47	13.7
Are the primary caregiver of clients with cardiovascular diseases in a clinical practicum		
Yes	192	56.1
No	150	43.9

### Cardiac knowledge and cardiac misconceptions

The mean score in the Cardiac Knowledge Test was 12.27/18 (SD 2.38), with the highest score being 17 and the lowest 2; the mode was 13 (19.6%). For the subscale scores, the mean score for knowledge related to risk factors was 3.2/4 (SD 0.75), diet 1.4/2 (SD 0.64), stress 4.65/7 (SD 1.17), and exercise 2.94/5 (SD 1.17). For misconceptions, the mean score for YCBQv1 was 6.98/18 (SD 2.84), with the highest score being 14 and the lowest 0; the mode was 7 (13.2%) (Refer to Table 2).

**Table 2:** Cardiac knowledge and misconceptions

Scale	Mean (SD)
Total number of correct answers in the Cardiac Knowledge Test	12.27 (2.38)
Knowledge related to risk factors	3.20 (0.75)
Knowledge related to diet	1.49 (0.64)
Knowledge related to stress	4.65 (1.27)
Knowledge related to exercise	2.92 (1.17)
Score for cardiac misconceptions	6.98 (2.84)

For YCBQv1, there were six items that more than half of the nursing students answered wrongly. They were: “Heart problems will definitely shorten your life whatever age you are”, “One of the main causes of heart diseases is stress”, “It is dangerous for people who have heart problems to argue”, “People who have a heart problem should always avoid stress”, “Rest is the best medicine for heart problems”, and “Angina is a kind of small heart attack”.

The relationships between the total and subscales of the Cardiac Knowledge Test and YCBQv1 was examined. The Pearson’s correlation coefficient was significant between YCBQv1, three subscales, and the overall knowledge score in the Cardiac Knowledge Test (*p* < 0.001) (Refer to Table 3).

**Table 3:** Bivariate Correlations between YCBQv1 and Subscales of the Cardiac Knowledge Test

Total	Risk factors	Diet	Stress	Exercise	Total Knowledge
Pearson’s Corr. coefficient	0.09	−0.24^a^	−0.20^a^	−0.26^a^	−0.33^a^
*p*-value	<0.001	<0.001	<0.001	<0.001	<0.001

^a^Correlation is significant at α = 0.01

A Chi-square test was used for additional insights into associations between demographics and misconceptions of cardiac diseases. There was a significant association between students who had a history of cardiovascular disease and the belief that it is dangerous for people with heart problems to argue; between students who had been the primary caregiver of family members and the belief that having heart problems is a sign that you have a worn-out heart; between students who had been the primary caregiver of clients and the belief that having heart problems is a sign that you have a worn-out heart; and students who had been the primary caregiver of clients during their practicum and the belief that the main cause of heart diseases is stress (Refer to Table 4). No significant association was noted between other demographic characteristics and misconceptions of cardiac diseases.

**Table 4:** Associations between demographic characteristics and misconceptions about heart diseases

Demographics	Misconceptions		Sig. (d.f. = 1)
Students who had a history of cardiovascular disease	It is dangerous for people with heart problems to argue	4.99	<0.05
Had been the primary caregiver for family members or relatives	Having heart problems is a sign that you have a worn-out heart	4.85	<0.05
Had been the primary caregiver for clients during a practicum	Having heart problems is a sign that you have a worn-out heart	4.63	<0.05
Had been the primary caregiver for clients during a practicum	One of the main causes of heart diseases is stress	7.89	<0.005

## Discussion

Although it is not uncommon for people suffering from heart disease to have poor knowledge and maladaptive beliefs about the disease, it is natural to assume that nurses would be better informed and have fewer cardiac misconceptions because of their professional training and qualifications.

In reviewing nursing curricula in Hong Kong, it should be noted that the Nursing Council of Hong Kong oversees all nursing syllabi to ensure that Registered Nurses have the required core competencies. The curriculum on cardiovascular nursing includes material on anatomy and physiology, common disorders of cardiac conditions, nutrition and dietetics, special investigations, therapeutic procedures, surgical and medical nursing management, and contemporary therapeutic agents and the implications for nursing [29]. The curriculum adequately prepares junior nurses to meet the demands that they will encounter in general clinical settings.

Our results indicated that a majority of the students had been the primary caregivers of patients during their practicum, and some them had been caregivers for family members or relatives. During the caregiving process, students could not avoid giving information to clients regarding knowledge of the disease and coping strategies. It is therefore crucial to ensure that senior-year nursing students are delivering up-to-date and accurate information to their clients.

The results of the study indicated that the students had good knowledge about the risk factors of cardiac diseases and the diet that those with cardiac diseases should follow. The results for stress and exercise were somewhat satisfactory. One study showed that patients who had had a myocardial infarction (MI) did not perceive exercise to be a mechanism for long-term sustained changes in behaviour. The health benefits of exercise should therefore be promoted to influence the intention to adhere to physical exercise [30]. Ideal forms of exercise following an MI include aerobic exercises, arm exercises, and strength training [31]. Unfortunately, some nurses had the misconception that rest is the best medicine for heart problems, which may cause them to overlook the benefits of being active during the rehabilitation period. Despite the abovementioned benefits of exercise, to ensure safe practices nurses need to be specific and familiar with the types and levels of exercise that should be pursued, instead of solely advising patients to increase their level of physical activity.

Our study showed a low correlation between knowledge of diet, stress, exercise, overall cardiac knowledge, and misconceptions. The findings could be considered surprising, as those students who achieved a higher score in the knowledge test should have had fewer misconceptions. Further education is therefore needed to help the students to better utilize their knowledge and be able to think critically to dispel their misconceptions. The educational strategies that could be employed include using case or problem-based learning focusing on cultural and personal beliefs to help to dispel myths held by students to be true, and to clarify why patients or nurses hold such misconceptions. Other approaches include using concept mapping to help students to connect theories, analyse various kinds of information, and develop their analytical ability by clear representations [32]. As students are not empty vessels to be filled by the expert knowledge of teachers, this pedagogy is considered effective for exploring knowledge and misconceptions through intellectual engagement, the recognition of differences, and the forging of connections to the wider world.

There were a number of items to which more half of our respondents gave incorrect answers. The respondents agreed that “Heart problems will definitely shorten your life whatever age you are”. This belief could be related to the literature stating that heart failure patients have a much-reduced quality of life due to their frequent use of healthcare facilities and to frequent hospital readmissions [33]. In 2015 the Daily Telegraph reported that “Suffering from heart disease, stroke and type two diabetes can knock 23 years off life and yet they are largely preventable for 8 out of 10 people” [34]. The National Health Services clarified that the newspaper used the general term “heart disease”, but the study that they were referring to specifically looked at people who had suffered from a heart attack (myocardial infarction). The study was published in the peer-reviewed *Journal of the American Medical Association* (JAMA). The above figure did not appear to have come from the results of the main study, as the figure on 23 years of lost life referred to men aged 40 with a medical history of stroke, diabetes, and heart attack [35]. The above piece of information could be misleading, as not every citizen or nurse would be able to gain access to research published in medical journals; as a result, the message could easily have been misinterpreted. With regard to the misconception that “One of the main causes of heart disease is stress”, our results corroborated those of two studies, which respectively found that 81.3% of Asian nurses, including graduate nurses and nursing students, held a similar misconception [7], and that South Asians living in Illinois believed that reducing stress is important in preventing coronary heart disease [36]. Although a number of previous studies have demonstrated that occupational, psychosocial, and marital stress are related to coronary heart disease [37, 38], it should be noted that the specific heart disease in question was coronary heart disease and not all kinds of heart diseases. Moreover, the association may be indirect, as work-related stress could be related to low levels of physical activity, a poor diet, and metabolic syndrome, which could increase the likelihood of developing heart diseases [39]. Nursing students should be reminded that they need to be cautious and critical when reading information from the media and from the literature, as the information requires interpretation and judgement needs to be exercised when acquiring new knowledge.

The other misconceptions included believing that “It is dangerous for people who have heart problems to argue”, “People who have a heart problem should always avoid stress”, and “Rest is the best medicine for heart problems”. Comparing this study with Lin’s study on nursing students, with exception of the item “People who have a heart problem should always avoid stress”, the respondents in the two studies demonstrated similar beliefs. A systematic review showed that exercise-based cardiac rehabilitation proved to be effective in restoring health and in reducing cardiovascular mortality and hospital admissions [40]. Yet the concept of the “sick role”, developed by Talcott Parsons, assumes that patients should be exempted from a list of social roles and responsibilities, or should neglect their usual duties to some extent [41]. If nurses instil those misconceptions in cardiac patients, the consequences could include patients who are unable to self-manage their condition, who live a sedentary life, and who are unable to re-establish their usual relationships with friends and family members. The goal of cardiac rehabilitation is to help patients to live a longer, better-quality life and to return to their normal daily activities; however, the myths conveyed by nurses could greatly affect the patients’ recovery and lead to unnecessary anxieties and complications. Our results echoed the finding in Lin’s study that most nurses agreed that “Angina is a kind of small heart attack”. It should be noted that this maladaptive belief could influence the patients’ lifestyle and worsen their angina [7]. It is true that unstable angina should be treated as an emergency and can lead to a heart attack. However, stable angina occurs when the heart needs to work harder, usually during physical exertion; the discomfort is manageable and can be relieved with rest [42]. It is important for nurses to understand the basics of the two kinds of angina, when they mostly occur, and the symptoms and possible triggers. Only then will they be able to differentiate between the symptoms and correctly interpret them, and to advise patients on treatments and lifestyle modifications. It is obvious that stable angina should not be regarded as a kind of small heart attack.

The results of the Chi-square tests demonstrated that a larger proportion of students with a history of cardiovascular disease agreed that “it is dangerous for people with heart problems to argue”. Similarly, those students who had been a primary caregiver for family members or clients with heart problems agreed that “having heart problems is a sign that you have a worn-out heart”. Having heart disease does not imply that one has a failing heart unless one is suffering from chronic heart failure, as heart failure can be disabling and can severely affect a person’s quality of life [43]. It is considered inappropriate to agree with a patient or to tell them that they have a worn-out heart, as the information could have detrimental effects on their rehabilitation and integration to normal life. Lastly, a larger proportion of students who had been a primary caregiver for clients agreed that “one of the main causes of heart diseases is stress”. The recent literature has established that prolonged exposure to community noise and traffic-related air pollution is associated with an increase in morbidity and mortality from respiratory and cardiovascular diseases. A recent systematic review revealed that air pollution can significantly increase the demands on the heart, which can lead to heart failure [44–46]. Excessive stress could be related to some form of heart disease, but would not be the main cause. Nursing students should be facilitated to critically seek meaning from knowledge and to relate concepts from various perspectives to real life situations.

This study presents a framework for designing the contents of cardiac nursing and is a starting point for promoting research on misconceptions held by undergraduate nursing students. If common misconceptions are not targeted in student learning, the increasing investment in theoretical knowledge may prove to be ineffective at dispelling incorrect beliefs held by the future nurses. The new paradigm of teaching should include inputs from both perspectives to help nursing students to critically use theoretical knowledge and rectify their misconceptions to pursue excellence in the working world.

### Limitations of the study

Some limitations should be noted when drawing conclusions from the findings of this study. Although the size of the sample in this study is considered large, the non-probability sampling from the three study universities reduced the rigour for representativeness. As for the questionnaire on cardiac knowledge, it is considered comprehensive in terms of exploring the various dimensions of knowledge, but it may not provide sufficient depth to tap into the various aspects of the nursing curriculum. Despite the limitations of our current study, it is of value in that it addresses the level of cardiac knowledge and misconceptions among nursing students. Our findings are relatively similar to recent relevant studies conducted in Southeast Asia. It would be very helpful to take a longitudinal approach to assess the improvement in students after the necessary knowledge on stress has been reinforced and the myths about cardiac health have been rectified.

## Conclusions

No previous study has been conducted with nursing students on knowledge and misconceptions about heart diseases. Our results show that nursing students had reasonable level of knowledge, but this is not necessarily an indication that they do not hold negative beliefs. Furthermore, associations were found between the students’ previous caregiving experiences and misconceptions. Nurses should be educated about risk factors, diet, exercise, and stress related to cardiac diseases, so that they will be able to educate patients in various clinical settings. Other than knowledge, nurse educators could incorporate the findings of the study into their courses, to better prepare nurses to understand misbeliefs about exercise and stress that could affect the patients’ journey to recovery.
